# Interpreting liver function markers in children on continuous renal replacement therapy for acute liver failure

**DOI:** 10.1007/s00431-025-06512-y

**Published:** 2025-10-11

**Authors:** Wun Fung Hui, Akash Deep

**Affiliations:** 1 Paediatric Intensive Care Unit, King’s College Hospital NHS Foundation Trust, London, UK; 2 Department of Women and Children’s Health, School of Life Course Sciences, King’s College London, London, UK

**Keywords:** Acute liver failure, Continuous renal replacement therapy, Hepatic markers, Kinetics, Children, Intensive care

## Abstract

Children with acute liver failure (ALF) often experience hyperammonaemia, fluid overload, metabolic derangements, and multi-organ failure. Continuous renal replacement therapy (CRRT) is commonly used for renal and non-renal indications, particularly in those with hyperammonaemia and hepatic encephalopathy. However, the non-selective nature of CRRT can result in the clearance of beneficial metabolites and liver-related biomarkers, which are important for monitoring disease progression or improvement. Therefore, understanding the kinetic principles governing molecular clearance during CRRT is crucial for accurate interpretation of liver biochemistry to inform clinical decision-making. By considering the kinetic properties of hepatic markers, including bilirubin, bile acids, liver enzymes, metabolites, waste products, inflammatory markers, albumin, and coagulation proteins, we describe the impact of CRRT on the plasma levels of these markers. In addition, we discuss various CRRT attributes, and the removal of these markers is also discussed. Metabolites and waste products, including ammonia, lactate, and urea, are the most useful markers to inform CRRT responsiveness, owing to their small molecular size, low degree of protein binding, and small volume of distribution; whereas bilirubin, bile acids, coagulation factors, and albumin remain true indicators of hepatic function and clinical condition. It should be noted that the overall plasma levels of any molecules reflect the balance between production and clearance, even for molecules with a high degree of CRRT removal. Awareness of these properties helps clinicians differentiate dialytic response versus genuine hepatic recovery or deterioration, guiding a more rational interpretation of clinical progression in ALF with liver transplant/spontaneous native recovery or mortality as the outcomes.

*Conclusion*: Understanding the kinetic behaviour of liver-related markers during CRRT is essential to distinguish between dialytic clearance and true changes in hepatic function. This knowledge enables more accurate clinical interpretation, guiding decision-making in children with acute liver failure and optimizing management strategies toward recovery or timely transplantation.

**What is Known:**

• *Children with acute liver failure often require CRRT for non-renal causes, including metabolic derangements, hyperammonemia, and multi-organ support.*

• *CRRT non-selectively clears both toxic metabolites and liver-related biomarkers, complicating the interpretation of hepatic function.*

**What is New:**

• *Accounting for the kinetic properties of liver markers during CRRT allows clinicians to distinguish dialytic clearance from true hepatic recovery or deterioration.*

• *Small molecules (ammonia, lactate, urea) might reflect CRRT responsiveness, whereas bilirubin, bile acids, albumin, and coagulation factors indicate liver function and clinical status.*

## Introduction

In critically ill children with acute liver failure (ALF), multi-organ failure ensues due to the release of cytokines by the failing liver. Though strictly not a liver assist device, continuous renal replacement therapy (CRRT) is frequently used as an extracorporeal support therapy to manage fluid overload, acute kidney injury, and metabolic derangements. In addition, it has also been increasingly employed for non-renal indications in ALF patients, including those with grade 2 or higher hepatic encephalopathy and hyperammonaemia [1]. CRRT acts by removing the toxic molecules or cytokines which cause multi-organ damage. However, due to the non-selective nature of the haemofilter, it also removes beneficial substances like electrolytes, nutrients, and drugs. The clearance of liver-specific molecules and metabolites that are used to monitor the progress of ALF by CRRT can therefore challenge the interpretation of liver biochemistry.

Laboratory tests for ALF typically indicate either liver dysfunction (e.g. bilirubin, serum protein, and coagulation tests) or the extent of liver injury (e.g. different liver enzymes). It is essential to understand both the natural history of hepatic markers and the impact of CRRT on biomarker levels. In ALF, a decreasing trend in liver markers can suggest liver regeneration, but it can also paradoxically indicate disease progression. For example, a sharp increase followed by a drop in alanine aminotransferase (ALT) and aspartate aminotransferase (AST) levels might suggest extensive hepatocellular injury and progressive deterioration, especially for those with ischaemic or drug-induced liver injury [2]. Bilirubin, which reflects hepatic dysfunction from impaired uptake, conjugation, or secretion, is typically moderately elevated in the early phase of ALF and progressively worsens as hepatic function deteriorates. Ammonia levels rise rapidly in ALF because its conversion to urea and glutamine primarily occurs in the liver. This makes ammonia an important prognostic marker, and hyperammonaemia enhances the risk of hepatic encephalopathy and cerebral oedema [3]. The liver is the main organ responsible for converting lactate to pyruvate via the Cori cycle, while the kidney is second to the liver in responsible for lactate metabolism [4]. In ALF, hyperlactatemia can result from the combined effect of multiple factors, including increased production due to circulatory failure and tissue hypoxia, reduced clearance due to hepatic failure and acute kidney injury, or mitochondrial dysfunction. Lactate levels rise early in ALF, and persistent hyperlactatemia has been increasingly recognized as an important prognostic marker [1, 2].

The trends of the liver biomarkers have a considerable impact on important clinical decisions. Falling ammonia levels, decreasing international normalized ratios, or lactate levels might make a clinician adopt a ‘wait and watch’ approach rather than urgently listing a patient for a liver transplant. At the same time, there is a significant variation in how clinicians institute CRRT for paediatric ALF, particularly concerning the timing of initiation and indications, dose, and anticoagulation. Therefore, clinicians should recognize the pitfalls of interpreting biomarker trends in the context of CRRT, avoiding false reassurance of the clinical conditions or overestimation of the disease severity.

The impact of CRRT on the trajectories of different molecules is different as each molecule has unique kinetic properties. In this manuscript, we summarize the key kinetic characteristics of liver-related molecules and discuss the relationship between molecular kinetics and CRRT specifics to support accurate interpretation of their levels for bedside decision-making.

## Kinetic considerations in ALF and CRRT

The effectiveness of clearance of any molecules during CRRT is determined by the interplay between the kinetic characteristics of the individual molecules and the specific CRRT characteristics applied (dose, modality, and type of filter used). The efficacy of removal can be described by the sieving coefficient (SC), which is the ratio of a solute’s concentration in the ultrafiltrate to its plasma concentration. A SC of 1 indicates full clearance by the haemofilter [5].

The SC of a molecule is primarily influenced by its molecular weight, degree of protein binding, and volume of distribution (Vd). A high SC is generally observed in molecules with a smaller molecular weight, lower degree of protein binding, and a smaller Vd [5, 6]. Unfortunately, SC is unavailable for many molecules due to the lack of kinetic studies; hence, a rational prediction of dialyzability is often necessary for common hepatic markers used in ALF. Table 1 summarizes the kinetic properties and clinical implications of the key metabolites, enzymes, bile acids, and inflammatory markers relevant to ALF.

**Table 1:** Kinetic properties of metabolites and molecules in liver failure

Molecule	Molecular weight (Da)	Protein binding	Volume of distribution	Half-life	Sieving coefficient	Implication
**Bilirubin**						
Unconjugated bilirubin	584.7	> 90%	9.9L	2–4 h	< 0.1	Unlikely to be removed by CRRT
Conjugated bilirubin	842.9	0–10%	14.8L	2–4 h	< 0.1	Limited clearance by CRRT
Delta bilirubin	582.6	100%	0.05L/kg*	12–21 days	~ 0*	Unlikely to be removed by CRRT
Free bilirubin	584.7	0%	–-	2–4 h	~ 1*	Theoretically removable by CRRT < 0.01% of unconjugated bilirubin
**Liver enzymes**						
ALT	54,000	N/A	N/A	47 h	0*	Intracellular enzyme and not removed by CRRT
ALP	86,000	N/A	N/A	7 days	0*	Membrane-bound enzyme and not removed by CRRT
AST	45,000 (monomer) 90,000 (dimers)	N/A	N/A	17 h	0*	Intracellular enzyme and not removed by CRRT
GGT	68,000	N/A	N/A	7–10 days	0*	Membrane-bound enzyme and not removed by CRRT
**Bile acids**						
Cholic acid	408.6	91.0%	3400L	2.8–4.3 days	–-	Unlikely to be removed by CRRT
Chenodeoxycholic acid	392.6	95.9%	1600L	45–96 h	–-	Unlikely to be removed by CRRT
Deoxycholic acid	392.6	98.2%	193L	4.1 h	–-	Unlikely to be removed by CRRT
Lithocholic acid	376.6	> 75%	-	17.8 h	0.01	Negligible removal by CRRT
Glycocholic acid	465.6	85.6%	2.6L	143 h	-	Unlikely to be removed by CRRT
Taurocholic acid	515.7	82.8%	-	2.8 days	0.08	Negligible removal by CRRT
Glycochenodeoxycholic acid	449.6	95.6%	-	-	-	Unlikely to be removed by CRRT
Taurochenodeoxycholic acid	499.7	97.2%	-	-	-	Unlikely to be removed by CRRT
**Metabolites/waste products**						
Ammonia	17.0	Minimal	0.4L/kg*	1–4 min	1*	Completely cleared by CRRT
Urea	60.1	~ 0%	0.6L/kg*	-	1	Completely cleared by CRRT
Creatinine	113	~ 0%	0.6L/kg*	3.2–9.7 h*	1	Completely cleared by CRRT
Lactate	90.1	~ 0%	0.45L/kg	20–60 min	0.96–1.0	Completely cleared by CRRT
**Inflammatory markers**						
C-reactive protein	115,000	N/A	0.05 L/kg*	19 h	~ 0*	Unlikely to be removed by CRRT
Procalcitonin	13,000	~ 0%*	0.2–0.3 L/kg*	20–24 h	0.07–0.24	Low level of removal by CRRT
**Proteins**						
Albumin	66,500	N/A	0.1–0.2 L/kg*	16–18 h (circulatory) 19–21 days (total body)	~ 0	Unlikely to be removed by CRRT
Fibrinogen	340,000	N/A	0.05 L/kg*	3–5 days	0*	Unlikely to be removed by CRRT
Factor V	330,000	N/A	0.05 L/kg*	12–36 h	0*	Unlikely to be removed by CRRT
Factor VIII	330,000	95–97%	0.07 L/kg	12 h	0*	Unlikely to be removed by CRRT
Factor IX	57,000	N/A	0.07–0.2 L/kg	29–43 h	0*	Unlikely to be removed by CRRT
von Willebrand factor	2,200,000	N/A	0.07 L/kg	9–15 h	0*	Unlikely to be removed by CRRT

*Estimated values

Kinetic properties that are generally associated with higher degree of removal during continuous renal replacement therapy include lower degree of protein binding (< 80%), lower volume of distribution (< 1 L/kg) and smaller molecular weight (< 1500 Da in CVVHD and < 20,000 Da in CVVH). Sieving coefficient of 0 denotes that the molecule is not filtered and 1 implies that the molecule is completely filtered

*ALP* alkaline Phosphatase, *ALT* alanine aminotransferase, *AST* aspartate aminotransferase, *CRRT* continuous renal replacement therapy, *GGT* gamma-glutamyl transferase, *N/A* not applicable

The modality of CRRT also affects removal efficiency. Continuous veno-venous haemodialysis (CVVHD) primarily clears small-sized molecules (< 500–1500 Da [Daltons]) through diffusion, whereas continuous veno-venous haemofiltration (CVVH) employs convection for the clearance of middle-sized molecules (1000–20,000 Da). In continuous veno-venous haemodiafiltration (CVVHDF), a combined effect is anticipated. Additionally, pre-dilution replacement fluid in CVVH may reduce the removal efficiency by diluting the patient’s blood [7]. Another important consideration is the dose of CRRT. We usually start with a higher dose (60 mL/kg/h) in ALF as compared to the conventional CRRT dose of 25–30 mL/kg/h in AKI or fluid overload [8]. The higher the CRRT dose used, the higher the removal of toxic metabolites, drugs, nutrients, and biomarkers. While other extracorporeal techniques like albumin dialysis or adsorption columns are increasingly used for ALF [9], their impact on hepatic markers falls outside the scope of this overview, as they are not the standard first-line treatments.

The following criteria predict a higher degree of CRRT removal (and a higher SC) for a given molecule after considering the above factors:

- Molecular weight < 1500 Da (CVVHD); < 20,000 Da (CVVH)
- Low protein binding (< 80%)
- Low volume of distribution (< 1L/kg)

## Specific groups of hepatic markers

### Bilirubin

Most forms of bilirubin, particularly unconjugated, conjugated, and delta bilirubin, are highly protein-bound and thus have negligible clearance by CRRT. Delta bilirubin, with a half-life of up to 21 days and 100% albumin binding, reflects cumulative cholestasis and may remain elevated even after resolution of acute liver injury. Conversely, free bilirubin (unbound fraction) is theoretically removable but represents only < 0.01% of total bilirubin, limiting its clinical impact.

### Liver enzymes

All liver enzymes (ALT, AST, alkaline phosphatase [ALP], and gamma-glutamyl transferase [GGT]) are large intracellular or membrane-bound proteins, with molecular weights ranging from 45,000 to 86,000 Da. Their SCs are estimated to be zero, indicating that they are unlikely to be filtered through CRRT membranes. Any decline in the levels of liver enzymes observed during CRRT likely reflects biological recovery, reduced cellular injury, or conversely progression of hepatic failure (for ALT and AST), instead of extracorporeal clearance.

### Bile acids

Bile acids have a high degree of protein binding (85–98%) and large volumes of distribution, often reflecting enterohepatic recirculation. Their pharmacokinetic properties make them poor candidates for CRRT clearance, with negligible sieving coefficients. This aligns with clinical observations where bile acid levels remain elevated despite renal support.

## Metabolites and waste products

Ammonia, urea, creatinine, and lactate meet the criteria for efficient clearance via CRRT with a low molecular weight, small Vd, minimal protein binding, and high sieving coefficients (~ 1). Owing to these characteristics, CVVHD or CVVHDF is the preferred modality for hyperammonaemia and hyperlactataemia requiring CRRT. Notably, ammonia (17 Da) has a very short half-life and is rapidly removed with effective CRRT, and its trend can serve as a surrogate marker for dialysis adequacy in hyperammonaemia [10]. It is worth noting that the overall levels of these metabolites also depend on the production rate [11]. Therefore, the failure of high-dose CVVHDF to reduce ammonia levels in ALF patients suggests that the rate of production exceeds the extracorporeal removal capacity. This lack of expected decrease in patients with no additional hepatic insult strongly indicates ongoing hepatic injury with uncontrolled ammonia production. Deep et al. have demonstrated that the inability to decrease ammonia after 48 h of starting CRRT was a poor prognostic sign reflecting increased ammonia production in severe paediatric ALF [8].

Lactate has a half-life of 20–60 min with favourable kinetic properties, making it an ideal candidate for CRRT removal. While its extracorporeal removal has been repeatedly demonstrated with high-dose CRRT, the CRRT clearance likely accounts for a small proportion of the total clearance, as the endogenous capacity of hepatic clearance is huge [11]. It should be emphasized that the trend of lactate levels also represents the balance between production and clearance, and improved lactate levels in the setting of ALF will be predominantly reflective of the combined effect of decreased production and/or improved endogenous hepatic and renal clearance, supplemented by CRRT clearance. Conversely, a rising trend or absence of reduction might indicate persistent shock or ongoing hepatic injury. Similarly, a rise in urea trends in children with ALF undergoing CRRT should prompt the clinician to seek possible causes such as increased protein intake, gastrointestinal bleeding, or worsening renal and hepatic function. This approach will be advised in conjunction with an adjustment of CRRT dosing and modality when facing an increasing trend of levels of these metabolites.

## Inflammatory markers and other proteins

C-reactive protein (CRP) and procalcitonin are frequently used as markers of infection and systemic inflammation in liver failure in which sepsis is a common complication and sometimes the cause of mortality. As CRP is primarily produced in the liver, its level may potentially be affected in ALF patients with compromised hepatic function. While CRP is not cleared by CRRT, procalcitonin has partial clearance, with significant removal occurring only with high-efficiency filters [12]. Caution is advised when interpreting serial procalcitonin trends in ALF patients on CRRT, particularly in liver failure induced or complicated by sepsis.

Albumin, fibrinogen, and clotting factors such as Factor V, VIII, IX, and von Willebrand factor are too large and/or protein-bound to be cleared by CRRT. A decline in these parameters reflects hepatic synthetic dysfunction rather than extracorporeal removal. Given their important roles in guiding blood product replacement, a correct interpretation of their levels is crucial during CRRT.

## Considerations in acute-on-chronic liver failure

Children with acute-on-chronic liver failure (ACLF) or those with chronic liver disease experiencing acute decompensation present additional complexity when undergoing CRRT. In these patients, the baseline synthetic dysfunction, altered protein binding capacity (due to hypoalbuminemia), and expanded extracellular fluid volumes in cirrhosis may impact the distribution and clearance of both endogenous molecules and administered drugs. Furthermore, the accumulation of inflammatory mediators and bile acids is more pronounced in ACLF, yet these are largely resistant to removal by conventional CRRT modalities. Clinicians should be particularly cautious when interpreting trends in bilirubin, bile acids, and coagulation markers, as these may reflect chronic hepatic dysfunction rather than acute deterioration or therapeutic response, and results must be interpreted within the broader context of hepatic reserve, transplant eligibility, and overall prognosis.

## Clinical implications

Although few studies have reported the dialyzability assessment and sieving coefficients for many of the molecules discussed above, a mechanistic understanding of molecular kinetics and CRRT principles can still provide guidance for rationally estimating the impact of CRRT on various biomarkers. This kinetic overview serves as a guide to distinguishing between biological and dialytic changes in liver function markers during CRRT. Clinicians should

- Avoid attributing declines in bilirubin or liver enzymes to CRRT removal
- Use ammonia, lactate, and urea as meaningful markers of CRRT performance
- Interpret inflammatory markers, especially procalcitonin, with caution
- Recognize that plasma proteins and coagulation factors are unaffected by dialysis
- Take note of the impact of CRRT modality and dose on trends of metabolites
- Consider ongoing production when an anticipated decline is absent for removable metabolites

## Conclusion

In children with ALF receiving CRRT, awareness of molecular kinetics is essential for accurate interpretation of liver function tests. While CRRT effectively removes small, unbound waste molecules, most liver-specific biomarkers, especially enzymes, bile acids, and clotting proteins, remain unaffected. A mechanistic understanding of these properties enables clinicians to separate artefactual changes from true hepatic recovery or deterioration, supporting safer and more informed care in the paediatric liver intensive care unit, including the decision for liver transplant listing.

## Data Availability

No datasets were generated or analysed during the current study.

## Abbreviations

ACLF: Acute-on-chronic liver failure
AKI: Acute kidney injury
ALF: Acute liver failure
ALP: Alkaline phosphatase
ALT: Alanine aminotransferase
AST: Aspartate aminotransferase
CRP: C-reactive protein
CRRT: Continuous renal replacement therapy
CVVH: Continuous veno-venous haemofiltration
CVVHD: Continuous veno-venous haemodialysis
CVVHDF: Continuous veno-venous haemodiafiltration
Da: Daltons
GGT: Gamma-glutamyl transferase
SC: Sieving coefficient
Vd: Volume of distribution
